# *Magnaporthe oryzae* infection triggers rice resistance to brown planthopper through the influence of jasmonic acid on the flavonoid biosynthesis pathway

**DOI:** 10.1111/1744-7917.13378

**Published:** 2024-05-15

**Authors:** Su Chen, Zhihuan Tao, Yanjie Shen, Rui Yang, Siyuan Yan, Zixu Chen, Bo Sun, Xiaofang Yang

**Affiliations:** ^1^ Precision Medicine Laboratory for Chronic Non‐communicable Diseases of Shandong Province, Institute of Precision Medicine Jining Medical University Jining Shandong Province China; ^2^ Key Laboratory of Insect Developmental and Evolutionary Biology, CAS Center for Excellence in Molecular Plant Sciences Institute of Plant Physiology and Ecology, Chinese Academy of Sciences Shanghai China; ^3^ College of Medical Engineering & The Key Laboratory for Medical Functional Nanomaterials Jining Medical University Jining Shandong Province China; ^4^ Institute of Bioengineering, College of Chemical and Biological Engineering Zhejiang University Hangzhou China

**Keywords:** flavonoid, jasmonic acid, *Nilaparvata lugens*, rice, rice blast

## Abstract

In agroecosystems, plants are constantly exposed to attack from diverse herbivorous insects and microbes, and infestation with one species may change the plant defense response to other species. In our investigation of the relationships among rice plants, the brown planthopper *Nilaparvata lugens* (Stål) and the rice blast fungus *Magnaporthe oryzae*, we observed a significant increase in the resistance of rice treated with rice blast to *N. lugens*, as evidenced by improved plant survival rates in a small population resistance study. Subsequent transcriptome data analysis revealed that the rice blast fungus can induce the expression of genes in the jasmonic acid (JA) and flavonoid pathways. Similar to the flavonoid pathway, the JA pathway also contains 2 types of genes that exhibit similar and opposite trends in response to *N. lugens* and rice blast. Among these genes, the *osjaz1* mutant and the *osmyc2* mutant were phenotypically confirmed to positively and negatively regulate rice resistance to *N. lugens* and rice blast, respectively. Subsequent mass spectrometry and quantification experiments showed that the exogenous application of methyl jasmonate (MeJA) can induce the accumulation of eriodictyol, naringenin and quercetin, as well as the expression of *OsF3H*, *Os4CL5* and *OsCHI* in the flavonoid pathway. This suggests a close connection between the JA pathway and the flavonoid pathway. However, *OsF3'H*, which negatively regulates rice resistance to *N. lugens* and rice blast, did not show increased expression. Phenotypic and molecular experiments confirmed that OsMYC2 can bind to and inhibit the expression of *OsF3'H*, thus revealing the mechanism of rice resistance to *N. lugens* after treatment with rice blast. These findings will deepen our understanding of the interactions among rice, *N. lugens* and rice blast.

## Graphical abstract

We propose a model in which the jasmonic acid (JA) signaling pathway and the flavonoid pathway can synergistically or independently regulate rice resistance to both the brown planthopper and rice blast. Specifically, rice blast infection can stimulate rice resistance to the brown planthopper by activating the flavonoid pathway through the JA signaling pathway. Using transcriptome, metabolome and molecular experiments, we identified that following rice blast treatment, rice exhibited a brown planthopper resistance phenotype. The subsequent elucidation of the underlying mechanism of this observed phenomenon enhances our comprehension of these intricate interactions.

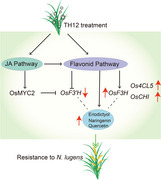

## Introduction

Rice stands out as one of the world's crucial food crops, with over half of the global population relying on it as their staple food source (Deng & He, 2023). Despite its paramount importance, rice faces significant challenges from pest and disease pressures, resulting in yield losses ranging from 24.6% to 40.9% (Savary *et al.*, 2019), thereby posing a substantial threat to food security in China. Notably, the brown planthopper *Nilaparvata lugens* (Stål), a hemipteran insect that feeds exclusively on rice, causes considerable damage by using its stinging mouthparts to extract sap from the phloem. This feeding leads to the yellowing and wilting of rice stems, and, in severe cases, to plant death (Zhang *et al.*, 2019). Concurrently, rice blast, a fungal disease caused by *Magnaporthe oryzae*, ranks as the primary disease affecting rice production globally, causing a 30% reduction in rice output and impacting the sustenance of 60 million people (Nalley *et al.*, 2016).

Plants have evolved a 2‐tiered immune system to prevent pathogen invasion: pattern‐triggered immunity (PTI), governed by pattern recognition receptors (PRRs), and effector‐triggered immunity (ETI), conferred by highly polymorphic resistance (R) proteins (Abramovitch *et al.*, 2006; Jones & Dangl, 2006; Couto & Zipfel, 2016; Boutrot & Zipfel, 2017). Through PTI/ETI processes, plants activate complex signaling systems via protein kinases and/or a calcium‐based signaling cascade, which stimulates the production of certain phytohormones and induces diverse defense mechanisms (Yuan *et al.*, 2021). The jasmonic acid (JA) signaling pathway is crucial for plant defense (Liu *et al.*, 2023). The JA signaling pathway, a major resistance regulator, responds to various stresses. The biosynthesis of JA is initiated with the release of *α*‐linolenic acid (*α*‐LeA, 18:3) from the lipid membrane by phospholipases (PLDs) in chloroplasts (Wasternack & Song, 2017). After a series of reactions catalyzed by 13‐lipoxygenase (LOX), allene oxide synthase (AOS) and allene oxide cyclase (AOC), 12‐oxophytodienoic acid (OPDA) is produced in chloroplasts (Wang *et al.*, 2019). OPDA is transported into peroxisomes and reduced by OPDA reductase 3 (OPR3) and then oxidized by acyl coenzyme A oxidase 1 (ACX1) to produce JA. JA is finally transported into the cytoplasm and conjugated to Ile by JASMONATE RESISTANT 1 (JAR1) to produce bioactive JA, JA‐Ile. Upon JA perception, the receptor F‐box protein CORONATINE INSENSITIVE 1 (COI1), a component of the S‐phase kinase‐associated protein 1 (Skp1)–Cullin–F‐box (SCF) E3 ubiquitin ligase, interacts with JA zinc‐finger inflorescence meristem (ZIM) domain (JAZ) proteins and targets them for degradation by the *26S* proteasome, thus releasing JA‐responsive transcription factors (TFs), such as MYC2, MYC3, MYC4, MYB21 and MYB24, and inducing the expression of JA‐responsive genes (Yan *et al.*, 2018). Silencing the JA synthesis pathway reduces rice resistance to chewing insects, but the infestation of rice with *N. lugens* increased the levels of salicylic acid (SA) and hydrogen peroxide, subsequently enhancing rice resistance to *N. lugens* (Qi *et al.*, 2011; Wang *et al.*, 2013; Zhou *et al.*, 2014; Zeng *et al.*, 2021). miR156 reduced rice resistance to *N. lugens* by elevating the JA content in rice (Ge *et al.*, 2018). *Nilaparvata lugens* feeding on rice upregulated genes in the JA pathway, increasing the level of JA. Silencing the *OsAOC* and *OsMYC2* genes in the JA pathway reduced the resistance of rice to *N. lugens* (Xu *et al.*, 2021). Increasing evidence suggests that the JA pathway is positively involved in the response of rice to blast fungus by facilitating the production of antimicrobial compounds (John, 2009; Riemann *et al.*, 2013; Taniguchi *et al.*, 2014). Transcriptome data indicate that *M. oryzae* can induce the expression of most genes in the JA signaling pathway (Wang *et al.*, 2023). The mutation of JA biosynthetic genes decreased rice resistance to *M. oryzae*, as observed in *osaoc1* and *osmyc2* mutants and in *OsCOI1* RNA interference (RNAi) lines (Qiu *et al.*, 2022). Additionally, the overexpression of JA‐responsive OsMYB TFs in rice significantly increases the resistance of rice to *M. oryzae* (Campos *et al.*, 2012), seen as the increased resistance of *OsMYC2*‐OE rice to *M. oryzae*. Therefore, the JA pathway plays a vital role in the response of rice to *N. lugens* and *M. oryzae*.

Jasmonic acid (JA) induces flavonoid accumulation in various fruits, such as apples (Fofana *et al.*, 2002; Xiao *et al.*, 2009), raspberries (Kruger *et al.*, 2001), blackberries (Wang *et al.*, 2008), blueberries (Huang *et al.*, 2015), strawberries (Perez *et al.*, 1997) and grapes (Flores *et al.*, 2015). Flavonoids, a major category of secondary metabolites in plants, significantly contribute to plant defense against biotic stresses such as pests and diseases (Jiang *et al.*, 2016). Comprising over 8000 metabolites in plants, flavonoids have a common diphenylpropane A backbone, with chalcones, orange ketones, flavanones, flavones, isoflavones, dihydroflavonols, colorless anthocyanins, anthocyanidin glycosides and proanthocyanidins being major subclasses. Of these, flavonoids and flavonols are the most abundant (Winkel‐Shirley, 2001; Havsteen, 2002; Kanno *et al.*, 2005; Kano *et al.*, 2005). Metabolomic analysis of flavonoid compounds from 14 plant species revealed 85 flavonoid compounds, with glycosylation being the predominant modification (Wang *et al.*, 2019). In the flavonoid synthesis pathway, chalcone synthase (CHS), chalcone isomerase (CHI), flavanone 3‐hydroxylase (F3H) and dihydroflavonol 4‐reductase (DFR) play pivotal roles (Kang *et al.*, 2019; He *et al.*, 2020). For instance, *OsF3H* positively regulates rice resistance to *N. lugens*, and O*sF3'H* negatively regulates resistance to both *N. lugens* and rice blast (Chen *et al.*, 2022). In wheat, *SbF3H* positively regulates resistance to anthracnose (Su *et al.*, 2021). In *Populus tremula*, the increased gene expression of *ANR*, *ANS*, *DFR*, and *F3H* could enhance the resistance of the plants to *Dothiorella gregaria* (Bai *et al.*, 2019). Naringenin, a precursor for the synthesis of numerous flavonoids, plays a protective role against UV light, pathogens and insects. The knockdown of uridine diphosphate glycosyltransferases (UDP)‐dependent glycosyltransferase (*Os07g32020*) in rice, leading to the increased accumulation of naringenin and sakuranin, enhances resistance to *N. lugens* (Yang *et al.*, 2021). Additionally, the concentration of quercetin was correlated with a greater toxic effect on *N. lugens* and with slowed *N. lugens* growth (Yang *et al.*, 2021). In wheat infected with *Fusarium graminearum*, exogenous spraying with kaempferol and apigenin increased resistance to downy mildew, affecting 789 metabolites, including various flavonoid compounds (Su *et al.*, 2021). Therefore, the flavonoid pathway may act downstream of the JA signaling pathway to regulate plant resistance to various biotic stresses.

A recent study has indicated the involvement of the JA pathway in rice resistance to *N. lugens* through regulating the flavonoid pathway (Liu *et al.*, 2023). However, it remains unclear whether the flavonoid and the JA pathways function effectively in an ecosystem where rice is attacked by multiple pathogens. In our study, we investigated the impact of *M. oryzae* infestation on rice resistance to *N. lugens* within the context of the rice−*N. lugens−*rice blast interaction. This approach aims to unravel the underlying mechanisms of resistance and contribute theoretical support for ecological management.

## Materials and methods

### Plant materials, insect populations and rice blast pathogen strain

The rice (*Oryza sativa* L.) varieties 9522, Nipponbare (Nip), Taichung Native 1 (TN1) and Zhonghua 11 (ZH11) were developed and maintained by our institute (CAS Center for Excellence in Molecular Plant Science, Shanghai, China). The *osjaz1* mutant (named *eg2‐1D*) was provided by Professor Dabing Zhang (Shanghai Jiao Tong University) in the 9522 background, isolated by screening an ethyl‐methane sulfonate‐induced mutant library (Cai *et al.*, 2014). The *osmyc2* mutant was obtained by CRISPR/Cas9 and maintained in the Nip background in the laboratory of Xuexia Miao (Institute of Plant Physiology and Ecology Chinese Academy of Sciences, Shanghai, China), with the CRISPR/Cas9 constructs produced in accordance with a previously reported protocol (Ma *et al.*, 2015). All plants were grown in fields of the experimental station at Songjiang Shanghai of CAS Center for Excellence in Molecular Plant Science or in a greenhouse held at 28 ± 2 °C with a 12‐h light/12‐h dark cycle and 70%–80% relative humidity.

The *N. lugens* populations used in this study, which were originally obtained from rice fields of the China National Rice Research Institute (Fuyang, China), comprised biotypes 1 and 2. They were maintained on susceptible rice cultivar TN1 plants in a climate‐controlled room at 26 ± 2 °C, with a 12‐h light/12‐h dark cycle and 80% relative humidity. The 2nd or 3rd instar nymphs were used for experiments.

The rice blast pathogen (*M. oryzae* TH12) was kindly provided by Prof. Zuhua He (CAS Center for Excellence in Molecular Plant Sciences, Shanghai, China).

### Plant inoculations

Plants were inoculated with the rice blast pathogen under field conditions using previously described punch and injection inoculation methods (Li *et al.*, 2020). ZH11, *osmyc2* mutant and Nip plants were sown in a field in Songjiang Shanghai. To prepare the inoculum, TH12 was grown on complete agar medium on plates for 2 weeks. Spores were collected by flooding the surface of the medium with sterile water. The spore concentration of the resulting suspension was adjusted to 1 × 10^5^ spores/mL before the punch and injection inoculations.

For the punch inoculation, detached rice leaves (6 cm in length) were cut in 3 places. Using a transfer pipette, 5 *μ*L of TH12 spore suspension was added to each cut site. The inoculated leaves were maintained in culture dishes containing 0.1% 6‐benzylaminopuricn (6‐BA) sterile water to ensure that they did not dry out. The culture dishes were placed in an incubator set at 28 °C. The typical symptoms of rice blast disease are a fusiform lesion with a gray center surrounded by brown necrotic tissue and a light‐yellow outer layer. The length of the lesion was measured using a ruler at 7 d after inoculation to compare differences in the resistance of the rice varieties to TH12. The test was replicated 15 times.

### Evaluation of N. lugens resistance

The resistance of individual rice seedlings or a small population of seedlings to *N. lugens* was evaluated according to a previously described method, which was slightly modified (Guo *et al.*, 2018).

To analyze individual plants, a single rice seedling was grown in a 7‐cm square pot with a plastic cage (6 cm in diameter; 30 cm in height). At the 3‐leaf stage, 15 2nd or 3rd instar *N. lugens* nymphs were released on each seedling. The test was replicated 6 times.

To evaluate small populations, approximately 30 seeds of each rice variety were sown in rectangular plastic boxes (length × width × height = 30 cm × 20 cm × 8.5 cm). At 7 d after sowing, the seedlings were thinned to 28 plants per plastic box. At 20 d after sowing, 5 2nd or 3rd instar *N. lugens* nymphs were added to each seedling. The test was performed in triplicate. For individual plant and small population experiments, the wild‐type (WT) plants of the *osjaz1* and *osmyc2* mutants are 9522 and Nip, respectively.

### Rice treated with TH12 followed by treatment with N. lugens

Ten “ZH11” seeds were sown in a plastic pot of 10 cm in diameter with a hole at the bottom. At the 2‐ to 3‐leaf stage, the seedlings were sprayed with 1 mL of TH12 spore suspension (1 × 10^6^ spores/mL) and ddH_2_O (as a control), respectively. After 24 h of treatment, 100 1st instar *N. lugens* nymphs were released on the seedlings. Each treatment was performed in triplicate.

### MeJA treatment

Ten “ZH11” seeds were sown in a plastic pot of 10 cm in diameter with a hole at the bottom. At the 2‐ to 3‐leaf stage, the seedlings were sprayed with 2.4 mL of methyl jasmonate (MeJA; 0 and 100 *μ*mol/L). The plants were collected 0, 0.5, 1, 2, 4, 6 and 9 h later, with 3 biological replicates per time point.

### Sample collection for the RNA‐seq analysis

At 45 d after sowing, 1 mL of TH12 spore suspension (1 × 10^5^ spores/mL) was injected into the leaf sheaths of ZH11 plants. The leaves were collected 0, 12 and 24 h later, with 3 biological replicates per time point. In total, 9 samples were collected.

### RNA isolation and RNA‐seq analysis

Total RNA was isolated from the 9 prepared samples using Invitrogen™ TRIzol™ reagent (Thermo Fisher Scientific, Waltham, MA USA), according to the manufacturer's instructions. The RNA was used to prepare libraries, which were sequenced on the BGISEQ‐500 analyzer at BGI (Shenzhen, China). The clean reads were assembled *de novo* as contigs using SOAPdenovo (https://github.com/aquaskyline/SOAPdenovo2). All reads were then realigned onto contigs according to the overlap relationship of the paired‐end reads and these contigs were then joined into scaffold sequences. Finally, the intrascaffold gaps were filled using the paired‐end extracted reads. The sequences that could not be extended in either direction were termed unigenes and were used in differentially expressed gene (DEG) analysis.

### RT‐PCR and RT‐qPCR assays

Total RNA was isolated from the collected samples using the Invitrogen™ TRIzol™ reagent. The RNA served as the template for synthesizing cDNA using the First Strand cDNA Synthesis Kit (TOYOBO, Osaka, Japan). Reverse transcription polymerase chain reaction (RT‐PCR) analysis was performed to examine the transcripts of the target genes. Reverse transcription quantitative polymerase chain reaction (RT‐qPCR) analysis was performed using the SYBR Green Real‐Time PCR Master Mix Kit (TOYOBO). A rice actin gene (*Os03g50885*) was used as the internal control for both the RT‐PCR and RT‐qPCR analyses. Details regarding all primers are listed in Table S1.

### Determination of the levels of eriodictyol, naringenin and quercetin

A standard curve of eriodictyol, naringenin and quercetin (YuanYe, China) was made using the following concentrations: 50, 10, 5, 1, 0.5 and 0.1 ng/mL. Rice seeds of ZH11 were hydroponically cultured for 3−4 d to accelerate germination before transplanting into pots. At 1 month after sowing, 50 mg of sturdy flag leaf taken from the same part of ZH11 plants treated with TH12 or with MeJA was sampled, ground in 300 *μ*L of acetonitrile and shaken for 1 h. Sodium chloride (20 mg) was then added, vortexed for 1 min and centrifuged at 4000 *g* for 10 min. The supernatant was centrifuged for another 5 min and then the supernatant was taken. The sample was placed in a weighing bottle, and the contents were determined using ultra‐performance liquid chromatography (UPLC) on a QTRAP 5500 instrument (SCIEX, Framingham, MA, USA). Each treatment was performed in triplicate.

### Dual luciferase analysis

The plasmid PHB‐35S‐MYC2‐Nos was transformed into *Agrobacterium tumefaciens* strain GV3101 to act as an effector. The reporter construct was prepared by cloning the *OsF3'H* promoter into the pGreenII0800‐LUC vector, and subsequently cotransformed with the helper plasmid pSoup19 into *A. tumefaciens* strain GV3101 to act as the reporter. The bacterial cultures from the experimental and control groups were infiltrated into the opposite ends of the same tobacco leaf (*Nicotiana benthamiana*). The leaves were collected after 3 d under long‐day white‐light conditions and infiltrated with 150 *μ*g/mL luciferin solution. Images were captured using a charge‐coupled device (CCD) camera 5 min later, and quantification was performed using the Dual‐Luciferase Reporter Assay System, according to the manufacturer's instructions (Promega, Madison, WI, USA). Three biological repeats were measured for each sample.

### Electrophoretic mobility shift assay

For the expression and purification of the DNA‐binding regions of OsMYC2, the Cy5‐labeled *OsF3'H* probe was amplified using 2 rounds of PCR. The DNA probes and proteins were co‐incubated in the reaction buffer, purified and incubated with the Cy5‐labeled probe at 37 °C for 20 min in electrophoretic mobility shift assay (EMSA) buffer (25 mmol/L HEPES pH 7.5, 40 mmol/L potassium chloride, 3 mmol/L dithiothreitol, 10% glycerol, 0.1 mmol/L EDTA, 0.5 mg/mL bovine serum albumin, 0.5 mg/mL poly‐glutamate). After incubation, the reaction mixture was electrophoresed on a 6% native polyacrylamide gel, and then labeled DNA was detected using a Starion FLA‐9000 instrument (Fujifilm, Tokyo, Japan).

### Subcellular localization

The conding sequences (CDSs) of the indicated genes were amplified by PCR and inserted into pC1300‐35S‐eGFP. The proteins fused with eGFP were transiently expressed in *N. benthamiana*, as described above. After staining with 4′,6‐diamidino‐2‐phenylindole (DAPI) to visualize the nucleus, the green fluorescent protein (GFP) and DAPI signals were examined 48 h later using a confocal microscope (LSM 880; Zeiss, Jena, Germany).

## Results

### Pre‐infestation of rice with TH12 confers resistance to N. lugens and alters gene expression in the JA and flavonoid biosynthesis pathways

**Fig. 1:**
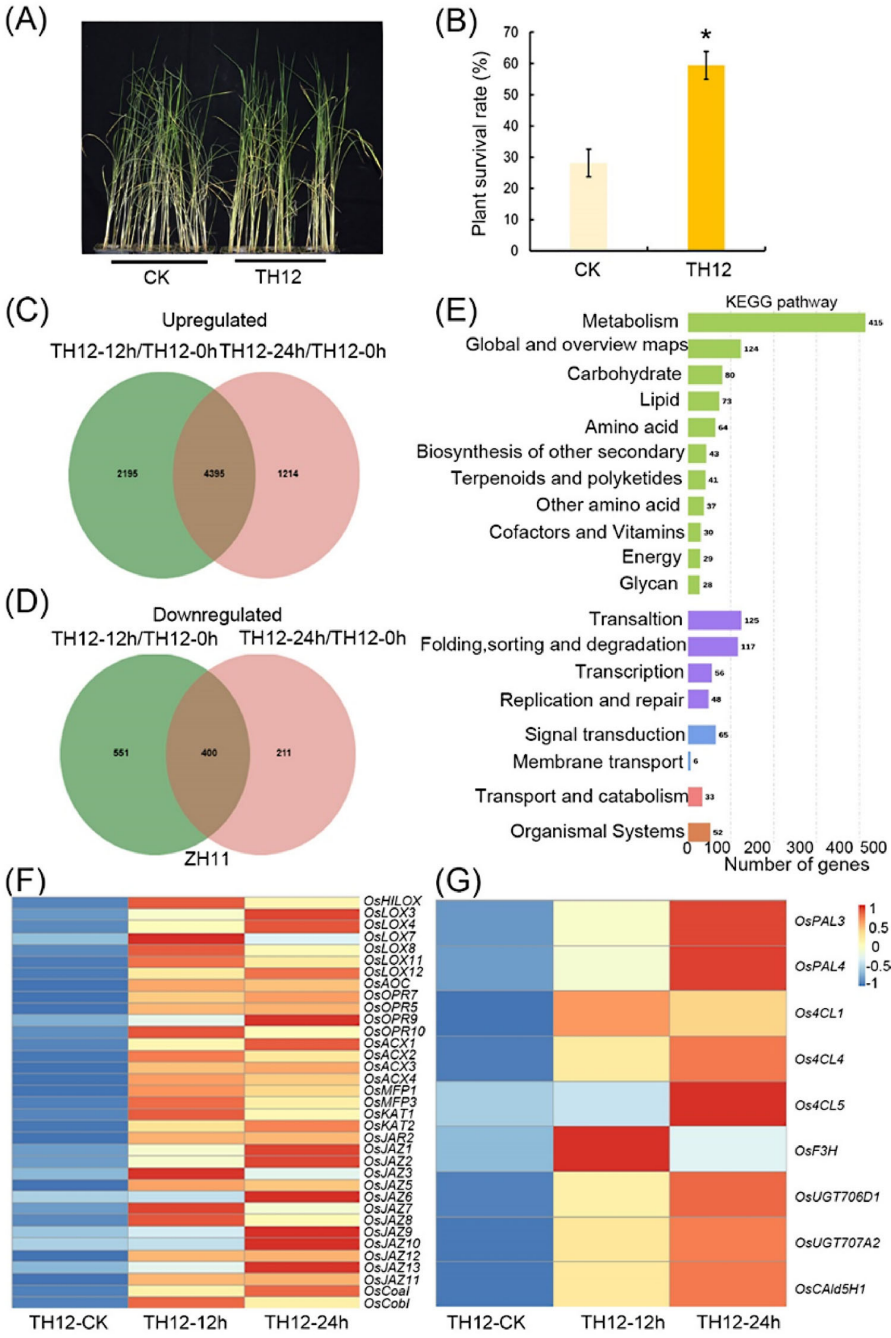
Phenotypic identification of rice infestation with *Nilaparvata lugens* after treatment with *Magnaporthe oryzae* strain TH12. (A) Phenotypes of rice infestation with *N. lugens* inoculated after treatment with TH12 for 24 h or not. (B) Survival rates of rice infestation with *N. lugens* inoculated after treatment with TH12 for 24 h or not. (C) Venn diagram of upregulated genes after treatment with TH12. (D) Venn diagram of downregulated genes after treatment with TH12. (E) Kyoto Encyclopedia of Genes and Genomes (KEGG) enrichment bar plot of upregulated genes in ZH11 plants after treatment with TH12. (F) Heat map of genes involved in the jasmonic acid (JA) pathway of rice infestation with TH12 for 24 h. (G) Heat map of genes in the flavonoid pathway of rice infestation with TH12 for 24 h. For the data in panel (B), significant differences were determined by Student's *t*‐test: *P < 0.05.

Initially, rice plants treated with TH12 exhibited enhanced resistance to *N. lugens* and an increased plant survival rate, compared with untreated rice plants (Fig. 1A, B). To decipher this phenomenon, we conducted an analysis of transcriptional changes using RNA‐seq on TH12‐treated samples. To ensure the reliability of our results, we calculated the Pearson's correlation coefficient (*r*) between sample replicates at each time point, revealing satisfactory intersample reproducibility for subsequent data analysis, with *r* values ranging from 0.747 to 0.994 (Fig. S1). Venn diagram analysis of the transcriptome data demonstrated that following 12 and 24 h of TH12 treatment, 6590 and 5609 genes were upregulated, respectively (Tables S2, S3). This resulted in a total of 4395 genes with upregulated expression (Fig. 1C). Additionally, analysis of genes with downregulated expression revealed 950 and 611 genes downregulated after 12 and 24 h of TH12 treatment (Tables S4, S5), totaling 400 genes with downregulated expression (Fig. 1D). Subsequent Kyoto Encyclopedia of Genes and Genomes (KEGG) enrichment analysis of the 4395 upregulated genes showed a higher enrichment of genes in metabolic pathways and signaling pathways (Fig. 1E). Further analysis through heat‐map visualization indicated the upregulation of most genes in the JA signaling pathway and some genes in the flavonoid pathway (Fig. 1F, G; Table S6). These results indicate that, ultimately, the JA and flavonoid pathway plays a key role in the process of TH12 inducing resistance to *N. lugens* in rice.

### Genetic analysis revealing consistent impacts of osjaz1 on N. lugens and TH12

**Fig. 2:**
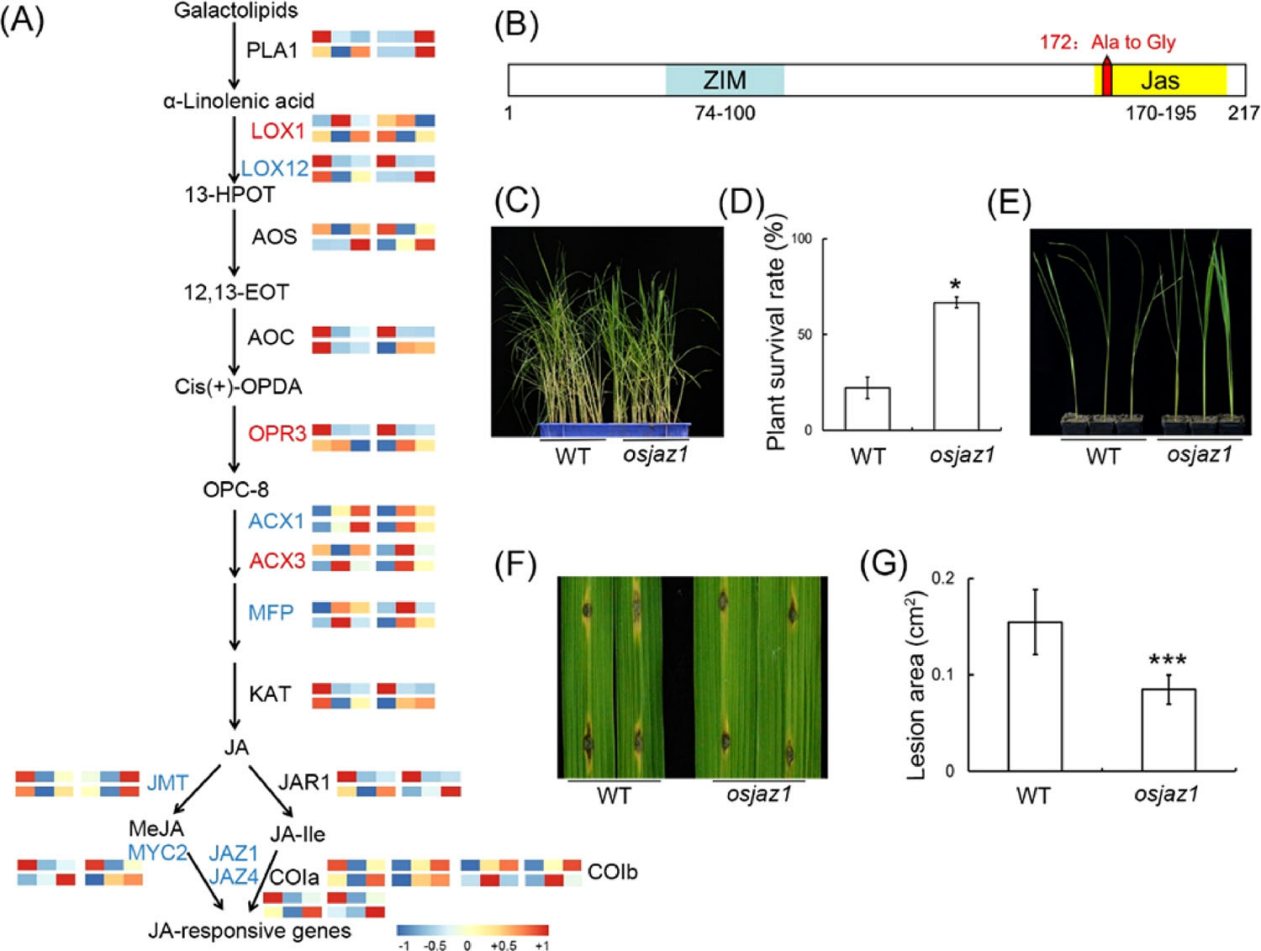
Genetics analysis revealing the consistent effects of *osjaz1* on *Nilaparvata lugens* and *Magnaporthe oryzae* strain TH12. (A) A simplified model of the jasmonic acid (JA) pathway and the heat map of the expression pattern of related differentially expressed genes (DEGs) in RHT and MDJ varieties at 0, 12 and 24 h after treatment with *N. lugens* and TH12. Each heat map can be divided into 4 regions. The upper left corner of the heat map indicates the results of 3 time points for the RHT variety after treatment with *N. lugens*; the lower left corner of the heat map indicates the results of 3 time points for the RHT variety after treatment with TH12. The upper right corner of the heat map indicates the results of 3 time points for the MDJ variety after treatment with *N. lugens*; the lower right corner of the heat map indicates the results of 3 time points for the MDJ variety after treatment with TH12. Genes in red had opposite expression trends after treatment with *N. lugens* and TH12 in the RHT and MDJ varieties; genes in blue words had consistent expression trends after treatment with *N. lugens* and TH12 in the RHT and MDJ varieties. (B) Sequence alignment of the *osjaz1* mutant with the wild type (WT). (C) Phenotypes of the WT and *osjaz1* plants after 7 d of infestation with *N. lugens* in a small population test. (D) Survival rates of WT and *osjaz1* mutant plants after 7 d of infestation with 5 2nd to 3rd instar *N. lugens* nymphs. Error bars represent the standard deviation (*n* = 3). (E) Phenotypes of WT and *osjaz1* plants individually infested with 20 2nd to 3rd instar *N. lugens* nymphs for 7 d. (F) Rice blast lesions on WT and *osjaz1* plants at 7 d after punch inoculation with isolate TH12. (G) Statistical analysis of the lesion area on WT and *osjaz1* plants at 7 d after inoculation with TH12. Error bars represent the standard deviation (*n* = 15). For the data in panels (D) and (G), significant differences were determined by Student's *t*‐test: *P < 0.05; ***P < 0.001.

Drawing on information from previously published studies (Chen *et al.*, 2022), we conducted an analysis of the expression patterns of key genes in the JA pathway following treatments with *N. lugens* and TH12. Our observations revealed that 7 genes (*OsACX1*, *OsJAZ1*, *OsJAZ4*, *OsJMT*, *OsLOX12*, *OsMFP* and *OsMYC2*) exhibited similar expression trends in the rice varieties MDJ (Mudanjiang) and RHT (Rathu Heenati) in response to both *N. lugens* and TH12 (Fig. 2A, with the gene names in blue; Table 1). Conversely, only 3 genes (*OsACX3*, *OsLOX1* and *OsOPR3*) displayed opposite expression trends in response to *N. lugens* and TH12 (Fig. 2A, with the gene names in red; Table 2). To validate our hypothesis, we generated *osjaz1* knockout lines using CRISPR/Cas9 technology (Fig. 2B). Results from subsequent infestation with *N. lugens* demonstrated that the *osjaz1* mutant exhibited greater resistance to *N. lugens* compared with the WT (Fig. 2C–E). Additionally, rice blast lesions were significantly smaller on the *osjaz1* mutant plants than on the WT plants (Fig. 2F, G). These findings suggest that *OsJAZ1* consistently influences rice resistance to both *N. lugens* and rice blast.

**Table 1:** Six genes in the jasmonic acid (JA) pathway with consistent expression trends in transcriptome analyses^†^

Gene	RHT	MDJ
*OsLOX12*	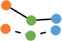	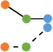
*OsACX1*	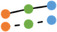	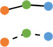
*OsMFP1*	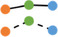	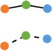
*OsJMT*	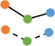	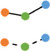
*OsJAZ4*	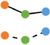	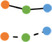
*OsJAZ1*	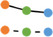	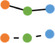

† Solid lines represent treatment with *Nilaparvata lugens*; dashed lines represent treatment with *Magnaporthe oryzae* strain TH12. Orange, green and blue dots represent 0, 12 and 24 h, respectively.

**Table 2:** Three genes in the jasmonic acid (JA) pathway with contrasting expression in transcriptome analyses^†^

Gene	RHT	MDJ
*OsLOX1*	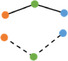	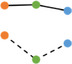
*OsOPR7*	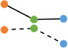	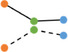
*OsACX3*	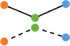	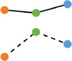

† Solid lines represent treatment with *Nilaparvata lugens*; dashed lines represent treatment with *Magnaporthe oryzae* strain TH12. Orange, green and blue dots represent 0, 12 and 24 h, respectively.

### MeJA induces the upregulation of most genes and compounds in the flavonoid pathway

**Fig. 3:**
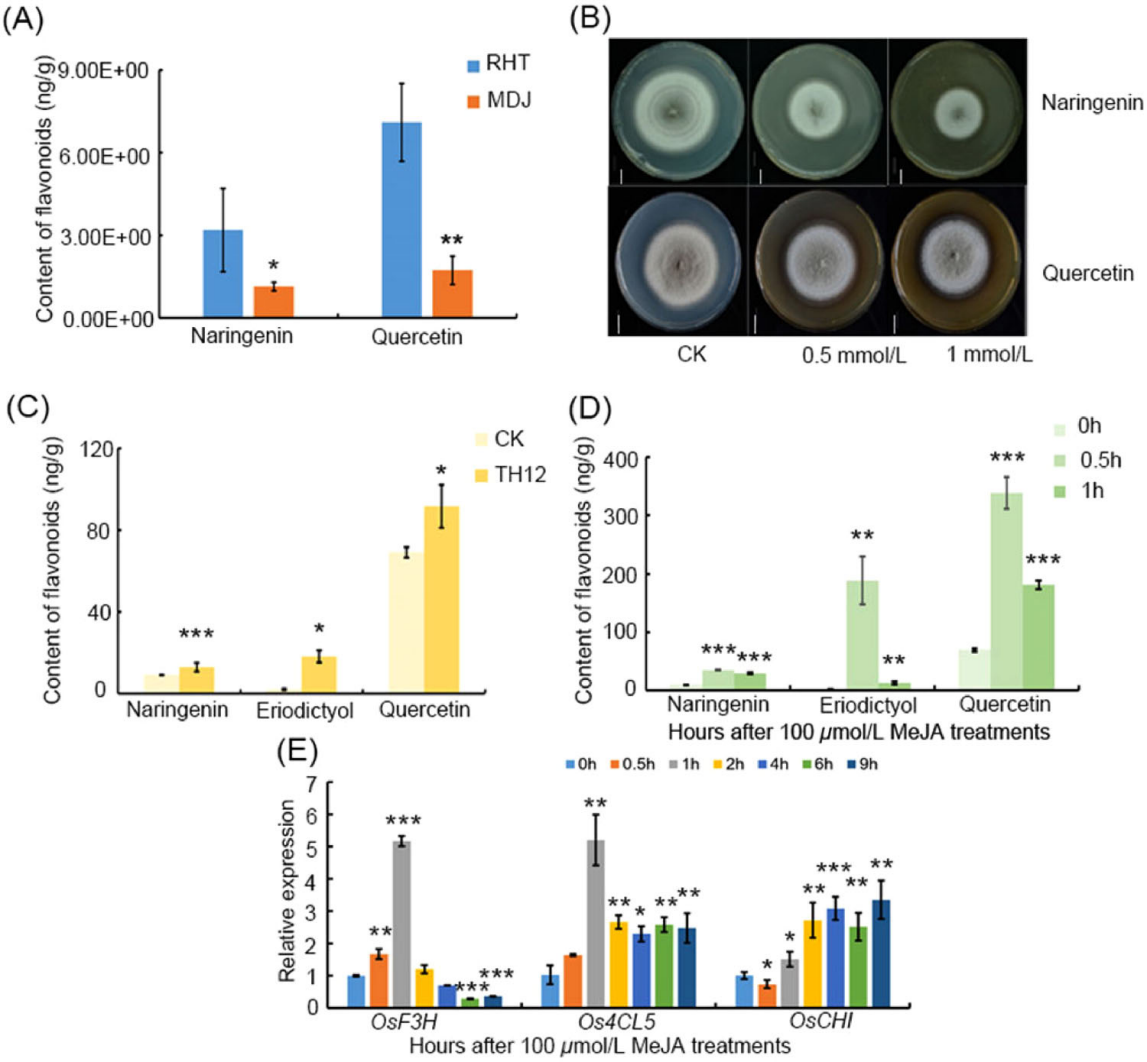
Methyl jasmonate (MeJA) induces the upregulation of most genes and compounds in the flavonoid pathway. (A) The contents of naringenin and quercetin in RHT and MDJ plants. (B) Growth of the *Magnaporthe oryzae* strain TH12 on CM plates supplemented with 0, 0.5 and 1.0 mmol/L naringenin and quercetin. The plates were incubated for 8 d. Scale bar: 1 cm. (C) The contents of naringenin, eriodictyol and quercetin after treatment with TH12 for 24 h. (D) The contents of naringenin, eriodictyol and quercetin after treatment with MeJA. (E) Upregulated genes in the flavonoid pathway after treatment with MeJA, determined by RT‐qPCR. For the data in panels (A)–(E), significant differences were determined by Student's *t*‐test: *P < 0.05; **P < 0.01; ***P < 0.001.

To investigate the correlation between the JA signaling and flavonoid pathways, we analyzed previously published metabolomic data (Chen *et al.*, 2022). Naringenin and quercetin levels were significantly higher in the resistant rice variety (RHT) compared with the susceptible rice variety (MDJ) (Fig. 3A). Subsequently, we cultured the fungal pathogen *M. oryzae*, strain TH12, on conditioned medium (CM) medium supplemented with 0, 0.5 or 1.0 mmol/L naringenin for 8 d. The elevated levels of naringenin resulted in a decrease in the growth rate of TH12 (Fig. 3B). Additionally, mass spectrometry findings demonstrated that the treatment of rice with TH12 or MeJA caused a corresponding increase in eriodictyol, naringenin and quercetin levels. The highest increase was identified after 0.5 h of MeJA treatment (Fig. 3C, D). We also treated rice plants with MeJA and conducted quantitative assays, demonstrating that MeJA treatments caused an upregulation in the expression of *OsF3H*, *Os4CL5* and *OsCHI* in the flavonoid pathway (Fig. 3E). This finding indicates that MeJA induces an increase in the levels of compounds and the expression of several genes in the flavonoid pathway.

### The expression patterns of *OsMYC2* under different treatments

**Fig. 4:**
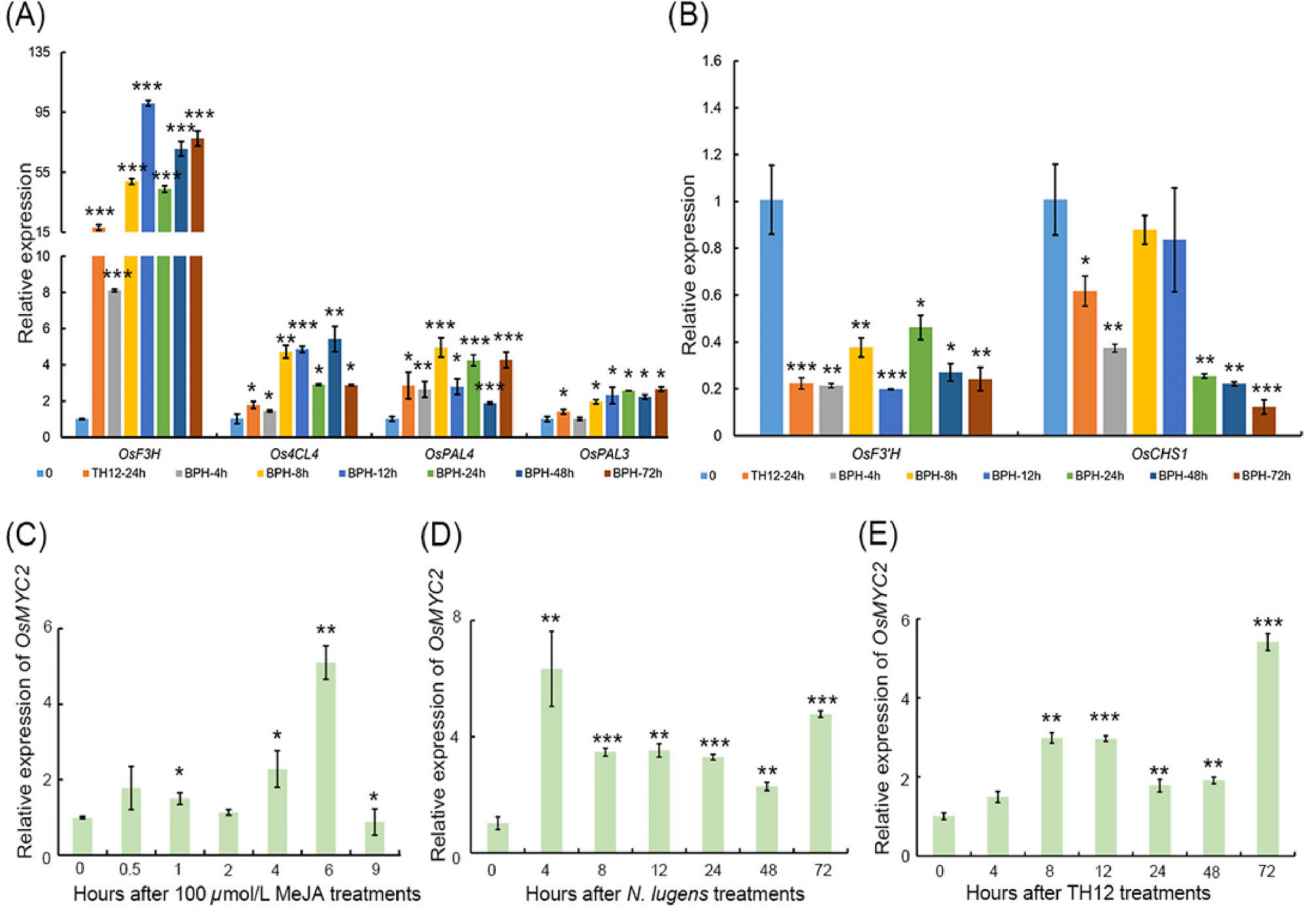
The expression pattern of *OsMYC2* under different treatments. (A) Upregulated genes expressed in the flavonoid pathway in rice plants treated with *Magnaporthe oryzae* strain TH12 for 24 h and then with *Nilaparvata lugens* until 72 h. (B) Downregulated genes expressed in the flavonoid pathway in rice plants treated with TH12 for 24 h and then with *N. lugens* until 72 h. (C) OsMYC2 expression in plants after treatment with 100 *μ*mol/L methyl jasmonate (MeJA), determined by RT‐qPCR. (D) OsMYC2 expression in plants at 72 h after treatment with *N. lugens*, determined by RT‐qPCR. (E) OsMYC2 expression in plants at 72 h after treatment with TH12, determined by RT‐qPCR. For the data in panels (A)−(E), significant differences were determined by Student's *t*‐test: *P < 0.05; **P < 0.01; ***P < 0.001.

In response to the above results, we administered TH12 to rice plants for 24 h and then exposed the plants to *N. lugens* until 72 h. We determined the expression levels of genes in the flavonoid pathway and identified that the expression of *OsF3H*, *Os4CL4*, *OsPAL4* and *OsPAL3* was upregulated, whereas the expression of *OsF3'H* and *OsCHS1* was downregulated (Fig. 4A, B). Rice blast‐induced JA signal transduction may encourage the accumulation of *N. lugens* resistance‐related flavonoids in rice and, given that OsMYC2 is a core transcriptional factor for JA signal transduction, we hypothesized that OsMYC2 might mediate TH12‐induced rice resistance to *N. lugens* through the regulation of flavonoid biosynthesis. For further confirmation, we cloned the CDS region of *OsMYC2*, eliminating the stop codon TGA, and ligated it into the pC1300‐eGFP vector. Via transient transformation of *N. benthamiana*, we found that the protein encoded by *OsMYC2* can localize to the nucleus, suggesting that MYC2 can act as a transcriptional regulator (Fig. S2A). Expression pattern analysis indicated that *MYC2* could respond to MeJA, TH12 and *N. lugens* treatments, underscoring the role of MYC2 in regulating rice resistance to *N. lugens* and rice blast (Fig. 4C–E).

### OsMYC2 has a transcriptional repressive effect on OsF3'H

**Fig. 5:**
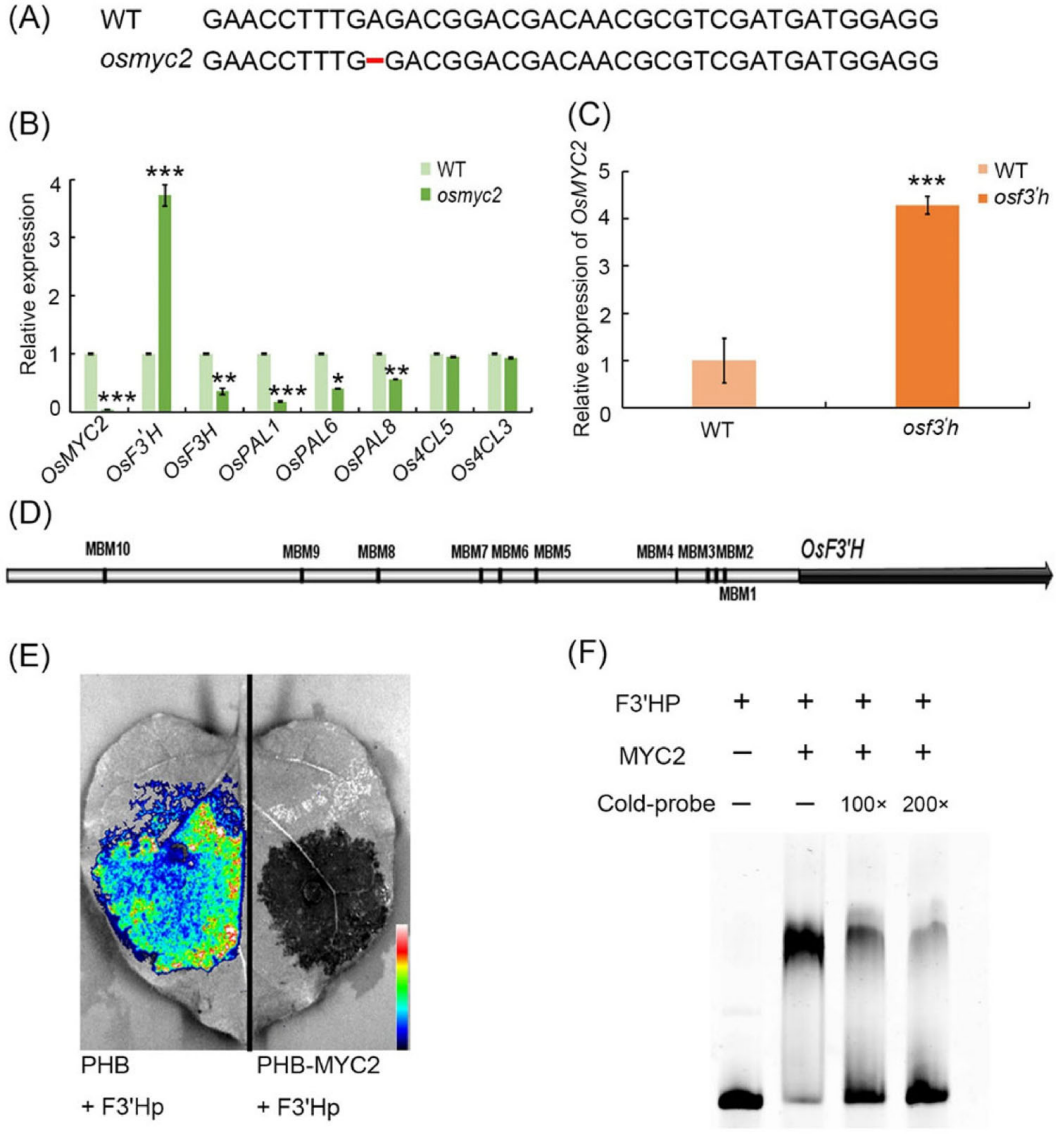
Analysis of the direct regulation of OsMYC2 on the *OsF3'H* gene. (A) Sequence alignment of the *osjaz1* mutant with the wild type (WT). (B) Gene expression in the flavonoid pathway in *osmyc2* mutants, determined by RT‐qPCR. (C) Expression of the *OsMYC2* gene in the *osf3'h* mutant, determined by RT‐qPCR. (D) Schematic representation of the 2‐kb *OsF3'H* promoter showing the positions of putative OsMYC2‐binding motifs (MBMs). (E) Dual luciferase assay to detect the activation of the *OsF3'H* promoter by OsMYC2 in *Nicotiana benthamiana*. (F) Electrophoretic mobility assay of OsMYC2 protein binding to the MBMs in (D). For the data in panels (B) and (C), significant differences were determined by Student's *t*‐test: *P < 0.05; **P < 0.01; ***P < 0.001.

Based on the above results, we used lab‐preserved *osmyc2* mutants (Fig. 5A), and we conducted an RT‐qPCR analysis of the *osmyc2* mutant. The majority of the genes in the flavonoid pathway were strongly downregulated in *osmyc2* mutant plants. However, the *OsF3'H* gene was upregulated (Fig. 5B). In addition, *OsMYC2* expression was upregulated in the *osf3'h* mutant plants (Fig. 5C). Through the transient transformation of *N. benthamiana*, we determined that the *OsF3'H* encoded protein was situated in the nucleus and cell membrane (Fig. S2A), and that rice treated with MeJA, TH12 and *N. lugens* exhibited opposite trends in the localization of OsF3'H (Fig. S2B‐D). Therefore, we speculate that *OsF3'H* may be the target gene of OsMYC2. To confirm our speculation, we assessed the promoter region of the *OsF3'H* gene and identified 10 OsMYC2‐binding motifs with the sequence CANNTG, in the 2‐kb promoter upstream of the “ATG” start codon (Uji *et al.*, 2016) (Fig. 5D). We then employed the Dual‐Luciferase® Reporter Assay System (Promega, Madison, USA) to verify the regulatory action of OsMYC2 on *OsF3'H*, and the results indicated that OsMYC2 can repress the expression of reporter LUC, driven by the *OsF3'H* promoter, demonstrating that OsMYC2 represses the transcription of *OsF3'H* (Fig. 5E). Furthermore, OsMYC2 exhibits no noticeable effect on reporter LUC driven by the *PAL1*, *PAL6* and *PAL8* promoters, the expression of which in the *OsMYC2* mutant is downregulated (Fig. S3). We examined the interaction of the recombinant OsMYC2 protein and a 40‐bp length of 5′‐Cy5‐labeled double‐stranded oligonucleotide possessing 3 CANNTG motifs proximal to the *OsF3'H* promoter utilizing EMSA. The DNA–protein complex migrated more slowly than the free DNA, suggesting the direct interaction of OsMYC2 with the labeled DNA. Ultimately, OsMYC2 bound directly to the promoter of *OsF3'H* to repress *OsF3'H* expression.

### The osmyc2 mutant rice has reduced resistance to N. lugens and rice blast

**Fig. 6:**
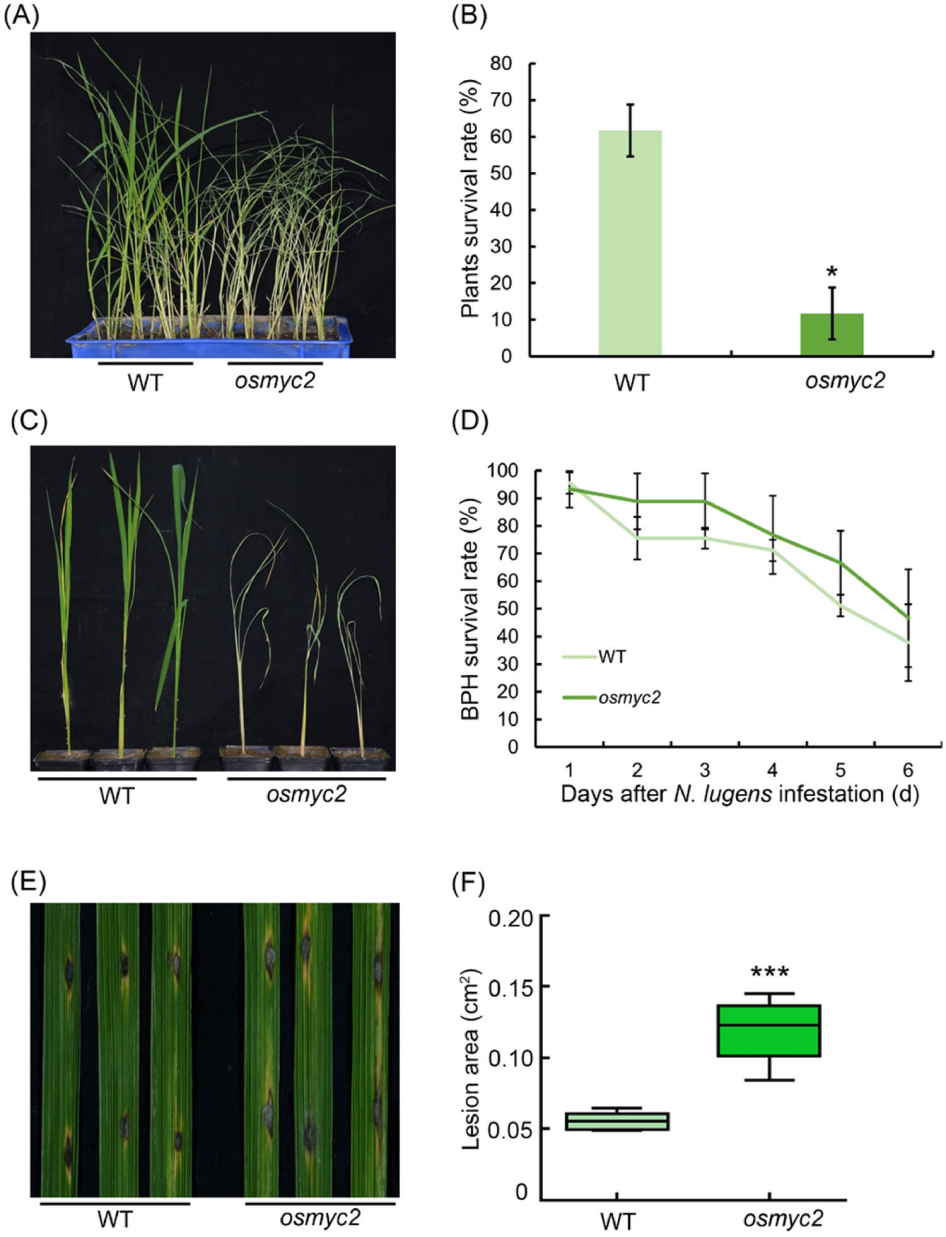
Identification of the phenotype of *osmyc2* mutants. (A) Phenotypes of the wild‐type (WT) and *osmyc2* plants after 7 d of infestation with *N. lugens* in a small population test. (B) Survival rates of WT and *osmyc2* mutant plants after 7 d of infestation with 5 2nd to 3rd instar *N. lugens* nymphs. Error bars represent the standard deviation (*n *= 3). (C) Phenotypes of WT and *osmyc2* plants individually infested with 20 2nd to 3rd instar *N. lugens* nymphs for 7 d. (D) Statistical analysis of *N. lugens* survival rates on WT and *osmyc2* plants after 8 d of infestation. Error bars represent the standard deviation (*n *= 6). (E) Rice blast lesions on WT and *osmyc2* plants at 7 d after punch inoculation with *Magnaporthe oryzae* strain TH12. (F) Statistical analysis of the lesion area on WT and *osmyc2* plants at 7 d after inoculation with TH12. Error bars represent the standard deviation (*n *= 15). For the data in panels (B), (D) and (F), significant differences were determined by Student's *t*‐test: *P < 0.05; ***P < 0.001.

We characterized the *osmyc2* mutant plants for *N. lugens* resistance across small populations and assessed the plant survival statistics. Our findings demonstrated that many *osmyc2* mutant plants died following inoculation with *N. lugens* for 1 week, whereas the WT plants remained alive (Fig. 6A). The survival rate of *osmyc2* mutant plants was significantly reduced compared with the WT plants (Fig. 6B). The WT plants and the *osmyc2* mutant plants were then characterized for individual resistance. After infection with the same number of *N. lugens*, the *osmyc2* mutant plants wilted and died, whereas the WT plants grew well (Fig. 6C). Concurrently, we assessed the number of *N. lugens* on the plants daily. Our findings demonstrated that the survival rate of *N. lugens* on *osmyc2* mutant plants was elevated compared with that on WT plants (Fig. 6D). These results indicate that the *osmyc2* mutant is more sensitive to *N. lugens* compared with the WT. Thereafter, WT plants and *osmyc2* mutant plants were inoculated with TH12, and spot counts were performed after 1 week. The results indicated that *osmyc2* mutant plants formed larger spots compared with WT plants (Fig. 6E, F), indicating that the *osmyc2* mutant is more sensitive to rice blast. Overall, the *osmyc2* mutant rice plants demonstrated reduced resistance to *N. lugens* and rice blast, and the *osf3'h* mutant rice plants demonstrated enhanced resistance to *N. lugens* and rice blast (Chen *et al.*, 2022). Therefore, based on the results displayed in Figs. 5 and 6, *OsF3'H* is a target gene of OsMYC2.

## Discussion

In agroecosystems, plants are consistently exposed to attack from diverse biotic stresses, including herbivores and pathogens, and any alterations in plants induced by one biotic stress may result in cascading effects on interactions with other biotic stresses. Systemic acquired resistance (SAR) is an essential part of the plant defense system, providing long‐lasting protection against a wide range of biotic stresses (Salwan *et al.*, 2023). For example, infestation with tissue‐chewing *Pieris rapae* larvae triggers a resistance response in *Arabidopsis thaliana* against subsequent attacks by the pathogens *Pseudomonas syringae* pv. tomato (Pst), *Xanthomonas campestris* pv. *armoraciae* (Xca) and turnip crinkle virus, and phloem feeding by *Myzus persicae* induces resistance to microbes (Vos *et al.*, 2007). Herbivorous insects and microbial pathogens share a partial overlap in the defense signaling pathways in plants, resulting in the development of resistance (Vos *et al.*, 2007). In the relationship among rice plants, *N. lugens* and rice blast fungus, when rice plants are infected with *N. lugens* the SA content increases, thereby enhancing the resistance of the rice plants to rice blast (Zhang *et al.*, 2020). Therefore, even though the areas affected by *M. oryzae* differ from those attacked by *N. lugens*, the infection of rice by *M. oryzae* may trigger systemic immunity in the rice plant.

In our study, pre‐infestation with *M. oryzae* TH12 conferred resistance to *N. lugens*. Similarly, infestation of rice seedlings with *Sogatella furcifera* inhibited infection by *M. oryzae* (Kano *et al.*, 2005). However, the mechanism of this phenomenon remains unclear. In this study, we determined that *M. oryzae* TH12 infestation can impact the expression of genes throughout the JA and flavonoid biosynthesis pathways (Fig. 1). We also identified some genes associated with JA pathways, salicylic acid pathway and ethylene pathway, which had contrasting and consistent expression trends after being treated with *N. lugens* and TH12 in different resistant rice varieties (Table S7), and the *osjaz1* mutant enhanced rice resistance to *N. lugens* and rice blast. Moreover, the JA signaling pathway plays an indispensable role in the modulation of rice resistance to *N. lugens* and rice blast (Fig. 2). Our findings demonstrated that MeJA can impact the expression of genes in the flavonoid biosynthesis pathway and the contents of eriodictyol, naringenin and quercetin in the flavonoid pathway in response to MeJA exposure (Fig. 3). According to recent research, similarly to sakuranetin, the levels of naringenin in the mutants of JA synthesis pathway‐related genes (*osmyc2*, *osaoc*, *osjar*, *oscoa1* and *oscoa2*) were significantly lower than those in WT plants. When the mutant plants are exposed to *N. lugens*, the naringenin levels are significantly increased, but this response is not observed for every flavonoid compound. For example, the concentration of eriodictyol‐7‐*O*‐*β*‐glucoside in *osmyc2*, *osjar* and *oscoa1* plants is significantly higher than that in WT plants. However, in *osaoc* and *oscoi2* plants, it is lower than in WT plants. After treatment with *N. lugens*, the concentration initially increases and then decreases over time (Liu *et al.*, 2023). Our analysis demonstrated that each compound has a higher concentration in JA synthesis pathway‐related gene mutants: the baseline levels differ, and the changes in these levels after treatment with *N. lugens* also differ. Therefore, when dealing with each different compound, analyzing the specific issues in detail is much better. Subsequently, the RT‐PCR results showed that the *OsF3H* and *OsF3'H* genes may have played different roles in the process of rice resistance to *N. lugens* induced by TH12 (Fig. 4). In addition, phenotypic and molecular experiments verified that OsMYC2 could bind to and inhibit the expression of *OsF3'H*. Given that *OsF3'H* negatively controls the resistance of rice to *N. lugens* and rice blast (Chen *et al.*, 2022), OsMYC2 can modulate the transcription of *OsF3'H* to control rice resistance against *N. lugens* under JA signaling (Figs. 5, 6). The results of previous studies have shown that the *osmyc2* mutant demonstrated reduced resistance to *N. lugens* in XiuShui11 (XS11) and to rice blast in ZH11 (Xu *et al.*, 2021; Qiu *et al.*, 2022; Jin *et al.*, 2023). Here, we show that the *osmyc2* mutant is more sensitive to *N. lugens* and rice blast in Nip. Thus, *OsMYC2* has a major role related to the regulation of rice resistance to *N. lugens* and rice blast. In the *osmyc2* mutant, unlike *OsF3'H*, the expression of many *OsPAL* genes and *OsF3H* is decreased. However, dual luciferase experiments showed that OsMYC2 has a distinct regulatory effect on these genes (Fig. S3). OsMYC2 has no apparent impact on *OsPAL6*, but it has an inhibitory effect on *OsF3H* and *OsPAL8*. We consider that the flavonoid biosynthesis pathway is complex, especially in plant resistance. For example, within a family, *OsPAL* genes may have similar functions, but their regulatory effects on *N. lugens* resistance can differ. In rice, 7 out of the 9 *OsPAL* genes are induced by *N. lugens*, among which *OsPAL6* and *OsPAL8* are specifically expressed in stems and leaf sheaths. The researchers overexpressed *OsPAL6* and *OsPAL8* in the susceptible variety 02428 background. Compared with the parental 02428 plant, the transgenic line overexpressing *OsPAL8* exhibited higher resistance to *N. lugens*, whereas the transgenic line overexpressing *OsPAL6* demonstrated even greater resistance to *N. lugens* than the parental plant (He *et al.*, 2020). This study demonstrates that the functions of genes in the phenylpropanoid pathway are influenced by their expression in various tissues and are triggered by different environmental stress factors, indicating functional specialization. As key genes in the flavonoid biosynthesis pathway, *OsF3H* and *OsF3'H* participate in various flavonoid biosynthesis processes, such as the production of dihydrokaempferol, dihydroquercetin, eriodictyol, kaempferol, quercetin and others. Therefore, whether MYC2 directly or indirectly regulates the expression of other genes and the composition of compounds in flavonoid biosynthesis still needs to be explored further. In summary, we propose a model in which the JA signaling pathway and the flavonoid pathway can synergistically or independently regulate rice resistance to *N. lugens* and rice blast. Rice blast infection can encourage rice resistance to *N. lugens* via the JA signaling pathway to activate the flavonoid pathway.

## Conclusion

**Fig. 7:**
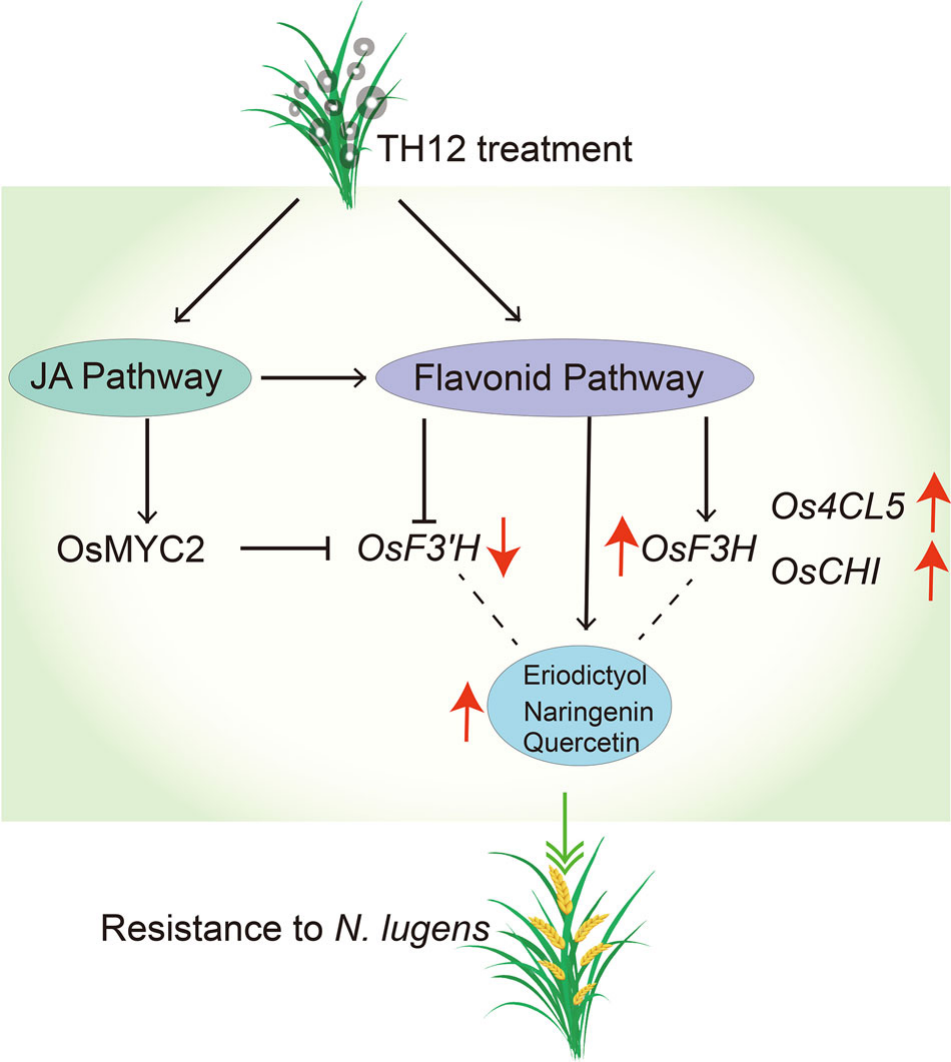
Schematic representation of rice showing the *Nilaparvata lugens* resistance phenotype after treatment with *Magnaporthe oryzae* strain TH12. Treatment with TH12 induces the expression of genes in the jasmonic acid (JA) signaling pathway, as well as the upregulation of the expression of most genes in the flavonoid pathway, and the elevation of eriodictyol, naringenin and quercetin levels in rice. Meanwhile, we also found that methyl jasmonate (MeJA) could also induce the expression of *OsF3H*, *Os4CL5* and *OsCHI*, but not *OsF3'H*, and increase the content of eriodictyol, naringenin and quercetin in the flavonoid pathway. Also, OsMYC2, the core transcription factor in the JA signaling pathway, can bind and repress the expression of *OsF3'H*, thus revealing the reason for the phenomenon of *N. lugens* resistance in rice after treatment with TH12.

In conclusion, we identified that following treatment with rice blast, rice exhibited a phenotype of resistance to the brown planthopper *N. lugens*, and using transcriptome, metabolome and molecular experiments, the underlying mechanism of this phenomenon was revealed. We further developed a model to deepen our understanding of these phenomena (Fig. 7).

Breeding rice varieties with resistance to multiple biotic stresses is a chief objective of rice breeders. Our research will assist in the investigation of the interactions among plants, insects and microbes to provide theoretical support for ecological governance.

## Disclosure

The authors declare that they have no competing interests associated with this work.

## Supporting information


**Table S1** All primer sequences used in this study.

**Table S2** Upregulated genes in ZH11 variety after TH12 12 h treatments.

**Table S3** Upregulated genes in ZH11 variety after TH12 24 h treatments.

**Table S4** Downregulated genes in ZH11 variety after TH12 12 h treatments.

**Table S5** Downregulated genes in ZH11 variety after TH12 24 h treatments.

**Table S6** Co‐upregulated genes in JA and flavonoid pathways after TH12 12 h and 24 h treatment.

**Table S7** Expression levels of salicylic acid pathway and ethylene pathway related genes after treatments with *N. lugens* and TH12.

**Fig. S1** Pearson correlation between transcriptome samples.

**Fig. S2** Localization of OsMYC2 and OsF3'H and the expression pattern of *OsF3'H* under different treatment.

**Fig. S3** Dual‐luciferase assay to detect the activation of the genes promoter, by OsMYC2 in *N. benthamiana*.
