# Supplementary figures and images for: Magnaporthe oryzae infection triggers rice resistance to brown planthopper through the influence of jasmonic acid on the flavonoid biosynthesis pathway
Source: Insect Sci. 2024 May 15;32(1):243–59. doi: 10.1111/1744-7917.13378 (PMC11824890; doi:10.1111/1744-7917.13378)

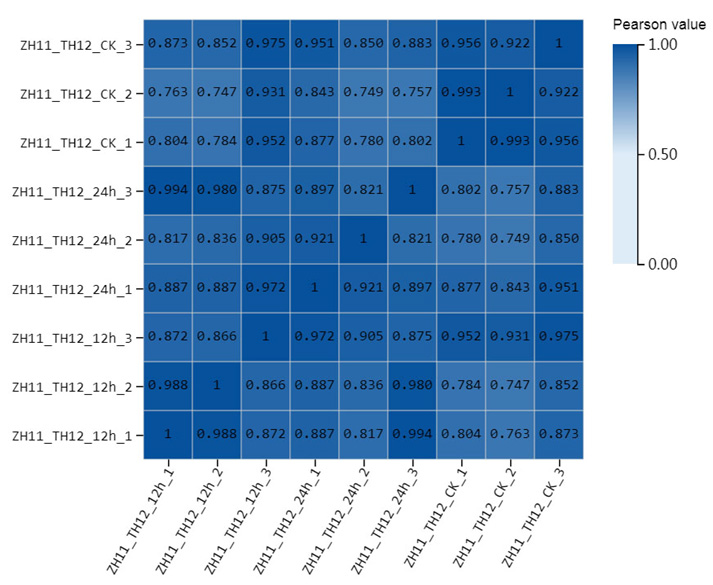

Supplement: Supplementary file 8 — Fig. S1 Pearson correlation between transcriptome samples. [file INS-32-243-s001.jpg]

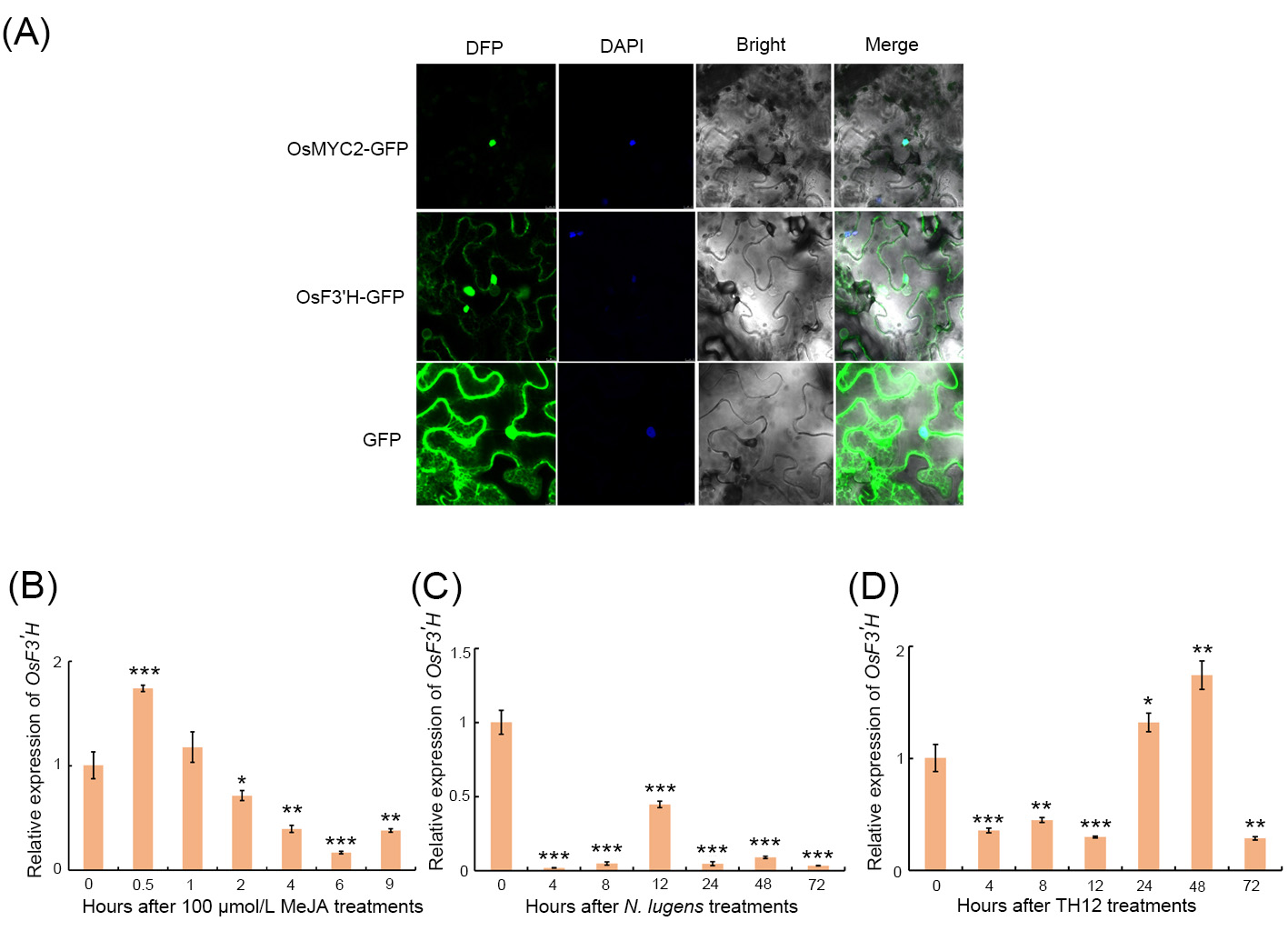

Supplement: Supplementary file 9 — Fig. S2 Localization of OsMYC2 and OsF3'H and the expression pattern of OsF3'H under different treatment. [file INS-32-243-s009.jpg]

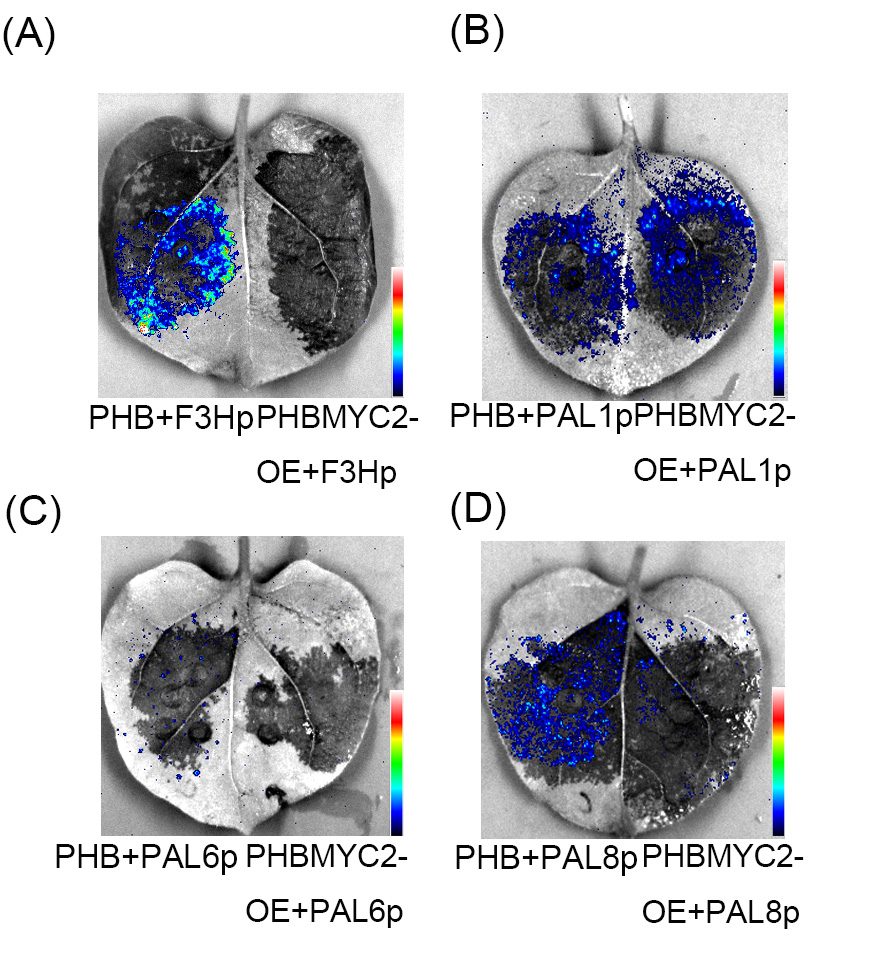

Supplement: Supplementary file 10 — Fig. S3 Dual‐luciferase assay to detect the activation of the genes promoter, by OsMYC2 in N. benthamiana. [file INS-32-243-s007.jpg]
